# Newly diagnosed Crohn’s disease, and hepatocellular and renal cell carcinoma in a bariatric surgery patient—dealing with the complexity of obesity-associated diseases: a case report and review of the literature

**DOI:** 10.1186/s13256-023-04111-9

**Published:** 2023-09-05

**Authors:** Lena Seidemann, Arne Dietrich

**Affiliations:** Department of Bariatric, Metabolic and Endocrine Surgery, Clinic for Visceral, Transplant, Thoracic and Vascular Surgery, University of Leipzig Medical Center, Liebigstr. 20, 04103 Leipzig, Germany

**Keywords:** Obesity-associated malignancies, Inflammatory bowel disease, Hepatocellular carcinoma, Renal cell carcinoma, Bariatric surgery, Case report

## Abstract

**Background:**

Bariatric surgery candidates commonly suffer from conditions that constitute the metabolic syndrome. But they also have a higher risk for autoimmune and malignant diseases. Obesity-associated comorbidities aside from the metabolic syndrome are often given insufficient attention in the clinical routine, including preoperative work-ups for bariatric surgery.

**Case presentation:**

We retrospectively report the case of a 65 years old Caucasian patient who was diagnosed with Crohn’s disease prior to, a hepatocellular carcinoma during, and a renal cell carcinoma post bariatric surgery. The relevance of these diseases for decision making in bariatric procedures and current recommendations for preoperative bariatric work-ups are discussed. In our case, the diagnosis of Crohn’s disease led to the performance of a sleeve gastrectomy instead of a Roux-en-Y gastric bypass and a previously unknown hepatocellular carcinoma was simultaneously removed by hepatic wedge resection.

**Conclusions:**

Preoperative endoscopy and imaging techniques can be valuable since surprising pre- and intraoperative findings can force the bariatric surgeon to change the initially planned operative strategy. But the diagnostic accuracy of abdominal ultrasound may be limited in bariatric surgery patients. With the expansion of bariatric surgery, the complexity of bariatric surgery patients is also likely to increase. However, with the appropriate awareness and strategies, bariatric surgery can be safely executed and even contribute to the treatment of severe comorbidities that exceed the metabolic spectrum.

## Background

Obesity is a complex disease state that is not only often accompanied by the classic comorbidities that constitute the metabolic syndrome, but is also associated with increased risks for autoimmune diseases and several types of cancer [1, 2]. Severe diagnoses may therefore be uncovered in patients presenting for bariatric surgery (BS) in their preoperative evaluation, unexpectedly during surgery or in the course of postoperative follow-up. We present the case of a patient who was scheduled for BS with a body mass index (BMI) of 47.7 kg/m^2^. Preoperatively known comorbidities were, among others, type 2 diabetes, obstructive sleep apnea (OSA), and a newly diagnosed Crohn’s disease (CD). Intraoperatively, a hepatocellular carcinoma (HCC) was discovered and in the postoperative course, a renal cell carcinoma (RCC) was found.

## Case presentation

A 65-year-old Caucasian male was referred to our bariatric surgery department with a BMI of 47.7 kg/m^2^. The patient had several obesity-associated comorbidities: type 2 diabetes, arterial hypertension, coronary heart disease with heart failure with New York Heart Association (NYHA) classification III, hepatic steatosis, hyperlipidemia, obstructive sleep apnea (OSA), and arthrosis in the lower limbs. Due to gastroesophageal reflux disease (GERD), we planned a laparoscopic proximal Roux-en-Y gastric bypass (RYGB) for the patient. However, gastric biopsies taken during routine esophagogastroduodenoscopy (EGD) prior to surgery revealed the presence of epithelioid cell granulomas suspicious for CD. An additional finding was a short segment Barrett’s esophagus without intraepithelial neoplasia. A subsequent colonoscopy confirmed the diagnosis of CD. Retrospectively, the patient reported diarrhea three times daily without blood admixtures or tenesmus. A systemic therapy with corticosteroids was initiated and surgery postponed. Six months later, after steroid weaning, the patient was asymptomatic concerning his CD. Weighing the risks for postoperative adverse events due to the inflammatory bowel disease (IBD) against the GERD and Barrett’s esophagus, a laparoscopic sleeve gastrectomy (LSG) was performed instead of the initially planned RYGB. The patient’s weight at time of surgery was 165 kg. His laboratory workup showed a normal kidney function and inconspicuous liver chemistry. An abdominal ultrasound had been performed earlier without noteworthy findings. Intraoperatively, a suspicious tumor with increased vascularization and a diameter of 2.5 cm was incidentally discovered at the margin of the steatofibrotically altered liver in segment 3. It was removed in toto with 1 cm margin by laparoscopic wedge resection and extracted via endobag. There were no further significant findings in the abdomen: no ascites, enlarged lymph nodes or peritoneal lesions, and no signs for portal hypertension. Thus, surgery was continued as planned: utilizing a 36 French bougie for calibration, a gastric sleeve was formed, thereby resecting an 800 ml portion of the stomach. A ventral repair of a 3 cm measuring hiatal hernia completed the procedure. Total operating time was 140 min. The postoperative course was uneventful and corresponded to the routine stay at our clinic. The pathological report of the liver tumor revealed a HCC stage pT1b G1 with tumor-free resection margins and no involvement of blood or lymph vessels. The surrounding liver tissue showed a grade 3 steatosis and stage 2 fibrosis, but no cirrhosis. Postoperative staging via chest computed tomography (CT) and abdominal magnetic resonance imaging (MRI) scans did not show any signs for metastatic spreading. However, an additional tumor, highly suspicious for malignancy, was discovered at the left kidney. Four months after LSG, a robot-assisted laparoscopic nephrectomy was performed. A completely resected clear-cell RCC stage pT1b G2 was confirmed pathologically. For both early stage malignancies, the patient was put on regular follow-up and did not receive systemic chemotherapy. He attended regular routine bariatric follow-ups, where he reported to be satisfied with his bariatric outcome. Total weight loss (TWL) was 23% and excess weight loss (EWL) was 49%. He never experienced any relapse of his CD, nor did he suffer from GERD symptoms. A control EGD was scheduled but had to be referred to an outpatient service due to capacity restrictions during the coronavirus disease 2019 (COVID-19) pandemic. The results of the examination could not be obtained. The severity of several of his obesity-related comorbidities significantly improved (see Table 1). Follow-up care after HCC and RCC was carried out at a clinic closer to the patient’s home in compliance with the German guidelines. Accordingly, abdominal MRIs were performed every 3 months in the first year, every 6 months in the second year, and thereafter once per year. Thoracic CT scans were done after 12 and 24 months. Three years after nephrectomy, when his weight had remained constant over half a year, the patient consulted with us to be planned for an abdominoplasty. Until that time, his malignant diseases had remained in full remission, but the 36 months follow-up imagings had not been performed yet. Unfortunately, during the preoperative work-up for plastic surgery, three small bilaterally localized pulmonary lesions were discovered by CT scan and were later histologically confirmed to be metastases of the RCC, in spite of the initially low tumor stage. Other signs for metastatic spreading were not discovered by positron emission tomography (PET)–CT. The patient was referred to his urologist for further oncologic care and did not present at our clinic thereafter.

**Table 1:** Overview on the patient’s obesity-associated comorbidities

	Diagnosis	Presurgery status/treatment	Postsurgery status/treatment
Resolved	Crohn’s Disease	Oral corticosteroid (budenoside); diarrhea 3× per day	Asymptomatic (medication stopped)
	GERD	Regular postprandial reflux symptoms; short segment Barrett’s esophagus	Asymptomatic (pantoprazole intake in combination with NSAIDs for arthrosis)
	HCC	pT1b G2	Complete resection; no recurrence during follow-up
	OSA	CPAP	Asymptomatic (CPAP no longer required)
Improved	Arterial hypertension	6 antihypertensives (spironolactone, torasemide, metoprolol, amlodipine, valsartan, hydrochlorothiazide)	2 antihypertensives (spironolactone, metoprolol)
	Type 2 diabetes	HbA1c 5.2%; no medication	HbA1c 4.7%
	Arthrosis	Regular NSAID and opioid intake (tilidine)	On-demand medication with NSAIDs
	Heart failure	NYHA Class III	NYHA Class II
Unchanged/no follow-up	Hyperlipidemia	Statin therapy	Unchanged
	Hepatic steatosis	25% of hepatocytes	No follow-up data available

*HbA1c* glycated hemoglobin, *OSA* obstructive sleep apnea, *CPAP* continuous positive airway pressure, *GERD* gastroesophageal reflux disease, *HCC* hepatocellular carcinoma, *NYHA* New York Heart Association Functional Classification, *NSAID* non-steroidal anti-inflammatory drug

## Discussion

BS has been proven to be safe and effective in treating obesity and its metabolic comorbidities and has become a main pillar in the multimodal treatment of patients with clinically severe obesity. However, health risks that are linked to excess body weight go beyond the metabolic syndrome. With the expansion of BS, the complexity of patients undergoing BS is also likely to increase.

### Patients undergoing bariatric surgery and inflammatory bowel disease

The prevalence of obesity is growing in patients with IBD similar to that of the general population [3]. Additionally, there is increasing evidence that obesity is a major environmental factor contributing to the onset and progression of autoimmune diseases, including IBD [2] and especially older onset CD [4]. But people with obesity seem to experience less severe disease courses of IBD [5]. In agreement with these observations, our patient was 65 years old at diagnosis and had only mild symptoms, which resolved quickly with steroid pulse therapy. Six months later, he already revisited our clinic with the persistent wish for a weight loss operation. Patients with IBD have a higher risk for perioperative complications [6]. However, a meta-analysis on patients with IBD undergoing BS found that LSG may result in fewer postoperative adverse events and thus be preferred over RYGB [7]. In the presented case, we decided to perform a LSG instead of the initially planned RYGB, and no postoperative complications occurred within the follow-up time of 3 years. Off note, the reflux symptoms also did not recur, presumably due to combined effects of weight loss and the performed hiatal hernia repair. Though certainly an exceptional case, in our patient the result of the routine preoperative EGD led to a change in the planned procedure. The importance and necessity of routine EGD prior BS is still a matter of debate. A systematic meta-analysis of 10,685 patients showed that in 82% EGD did not change the surgical plan and therefore questions its justification [8]. American guidelines recommend preoperative EGD only for symptomatic patients, while in Europe it is recommended for all bariatric patients [9]. Considering the highly elective setting of BS, our center shares the opinion that preoperative EGD should be exercised routinely.

Concerning CD symptom control, BS can lead to significant improvement, including the achievement of complete remission [3], as was also the case in our patient. Several reports have concluded that BS is feasible and safe in patients with IBD, at least on short-term follow-up [10, 11]. Nonetheless, post-bariatric relapse or de novo CD and severe disease-specific postoperative complications (for example, formation of fistulae) have also been reported [6]. Therefore, further studies, especially with a prospective design and longer follow-up, are desired to elucidate the role of BS for patients with IBD. Until then, thorough patient selection is needed, although admittedly precise selection criteria have yet to be defined.

### Patients undergoing bariatric surgery and metabolic dysfunction-associated steatotic liver disease

Metabolic dysfunction-associated steatotic liver disease (MASLD) is on the verge of becoming the main reason for end-stage liver disease [12]. It is a major risk factor for HCC, and alarmingly, MASLD-HCC tends to develop at earlier stages in non-cirrhotic livers, as was the case here [13]. This hinders HCC surveillance, which is so far aimed at patients with cirrhosis. Another report describes two cases of unexpectedly discovered HCCs during BS [14]. The other group also performed laparoscopic wedge resections and completed the planned bariatric operation. Several studies have shown that BS lowers the risk of HCC and other major adverse liver outcomes [15–17] and can lead to histological regression in metabolic dysfunction-associated steatohepatitis (MASH) and fibrosis [18]. Though BS can be safely performed in patients with cirrhosis, conforming studies mostly report on patients with mild stages (predominantly Child–Pugh A) [19]. Postoperative morbidity and mortality are increased, but are lower after SG than RYGB or other procedures [20]. Therefore, an intraoperative encounter of a cirrhotic liver can force the bariatric surgeon to question the initially planned operative concept. In our case, the liver had a steatofibrotic aspect, but was not full-stage cirrhotic nor were there any signs of portal hypertension. Otherwise, a cancellation of the bariatric procedure could have been the consequence, since morbidity and mortality of patients with portal hypertension are considerably increased [20]. For our patient, LSG was already planned due to the IBD and did not cause any postoperative reduction of the liver function.

### Patients undergoing bariatric surgery and other malignancies

Besides HCC, a whole group of malignancies is now confirmed to be obesity-associated; among others, adenocarcinomas of the gastrointestinal tract, cancer of the breast, endometrium, thyroid, and kidney [21]. For RCC, our patient exhibited several further risk factors: hypertension, a history of smoking (although he had quit several years prior surgery), and a frequent use of analgesics due to arthrosis. He had undergone abdominal ultrasound preoperatively, but the RCC diagnosis was only made on staging MRI for the HCC. In line with this incident, a case series questions the sensitivity of preoperative ultrasonography for RCC detection in bariatric patients [22]. All in all, the number of bariatric patients with a history of malignancy or a malign diagnosis after BS will increase [1]. Unexpected intraoperative discoveries of tumors suspicious for malignancy seem to be rare. Gagné *et al*. found only two cases in the retrospective analysis of their cohort of 1566 patients [23]. Considering the alarming cancer risk profile of bariatric patients, a question is if the implementation of a routine abdominal CT or MRI in the preoperative workup would be advisable. On the other hand, CT or MRI scans can sometimes not be performed because of extreme body measurements.

## Conclusions

BS can be safely executed in patients with Crohn’s disease and malignancies. In the best case scenario, it can resolve several problems at once and reduce the risks for further comorbidities. With the increasing numbers of patients seeking BS, a stronger focus should be placed on the complexity of obesity-related disorders that exceed the metabolic spectrum.

## Data Availability

The authors confirm that the data supporting the findings of this study are available within the article.

## Abbreviations

BS: Bariatric surgery
CD: Crohn’s disease
HCC: Hepatocellular carcinoma
OSA: Obstructive sleep apnea
RCC: Renal cell carcinoma
BMI: Body mass index
GERD: Gastroesophageal reflux disease
RYGB: Roux-en-Y gastric bypass
EGD: Esophagogastroduodenoscopy
IBD: Inflammatory bowel disease
LSG: Laparoscopic sleeve gastrectomy
CT: Computed tomography
MRI: Magnetic resonance imaging
TWL: Total weight loss
EWL: Excess weight loss
PET: Positron emission tomography
MASLD: Metabolic dysfunction-associated steatotic liver disease
MASH: Metabolic dysfunction-associated steatohepatitis
